# Case Report: Uncommon co-occurrence of different renal histopathological entities in a patient with multiple myeloma and lymphoplasmacytic lymphoma

**DOI:** 10.3389/fonc.2025.1672690

**Published:** 2025-10-07

**Authors:** Marco Talarico, Gisella Vischini, Simona Barbato, Roberta Restuccia, Simone Masci, Ilaria Sacchetti, Enrica Manzato, Stefano Ghibellini, Michele Puppi, Miriam Iezza, Ilaria Rizzello, Lucia Pantani, Paola Tacchetti, Enrica Borsi, Vincenza Solli, Carolina Terragna, Claudio Agostinelli, Gaetano La Manna, Michele Cavo, Elena Zamagni, Katia Mancuso

**Affiliations:** ^1^ IRCCS Azienda Ospedaliero-Universitaria di Bologna, Istituto di Ematologia “Seràgnoli”, Bologna, Italy; ^2^ Dipartimento di Scienze Mediche e Chirurgiche, Università di Bologna, Bologna, Italy; ^3^ Nephrology, Dialysis and Renal Transplant Unit, IRRCS Azienda Ospedaliero-Universitaria, Bologna, Italy; ^4^ Hematopathology Unit, IRCCS Azienda Ospedaliero-Universitaria di Bologna, Bologna, Italy

**Keywords:** multiple myeloma, lymphoplasmacytic lymphoma, acute kidney injury, renal damage, renal biopsy

## Abstract

In patients with plasma cell dyscrasias presenting with kidney injury, renal biopsy usually displays the presence of a single histological damage. However, the coexistence of heterogeneous damages has been occasionally described and is associated with worse renal outcomes. In this report, we describe the case of a patient with a concomitant diagnosis of multiple myeloma and lymphoplasmacytic lymphoma, presenting with acute kidney injury and with renal biopsy revealing the unexpected concurrent presence of several different renal damages, who achieved a good hematologic response and dialysis independence after anti-myeloma therapy.

## Introduction

Plasma cell (PC) dyscrasias and other lymphoproliferative disorders may be associated with heterogeneous types of renal involvement. The most frequent cause of kidney failure in patients affected by multiple myeloma (MM) is represented by light chain cast nephropathy (LCCN), which is commonly associated with high serum-free light chain (sFLC) and monoclonal proteinuria and is regarded as a myeloma-defining event (MDE) (1). Conversely, light chain amyloidosis (AL) and other monoclonal gammopathies of renal significance (MGRS) are generally related to underlying small clones (2, 3). Each condition presents distinctive histological features and may be associated with specific clinical and laboratory findings. In the majority of cases, renal biopsy reveals the presence of a single renal damage, although the coexistence of different patterns in the same patient has been occasionally described, with the combination of LCCN and light chain deposition disease (LCDD) being reported as the most common (4–8). The pathogenic mechanisms of renal injury are heterogeneous and have been classified as either direct or indirect based on the presence or absence of monoclonal immunoglobulin in the kidneys. These mechanisms involve physicochemical properties of the monoclonal immunoglobulin, immunological mechanisms, and features of the renal microenvironment (2).

Herein, we report on the case of a patient diagnosed with both MM and lymphoplasmacytic lymphoma (LPL) unexpectedly associated with the concurrent presence of multiple morphological renal damages, leading to dialysis-requiring acute kidney injury (AKI) as the main onset feature, and who achieved a good hematologic response and dialysis independence after anti-myeloma therapy.

## Case description: clinical presentation and diagnostic assessment

**Figure 1:**
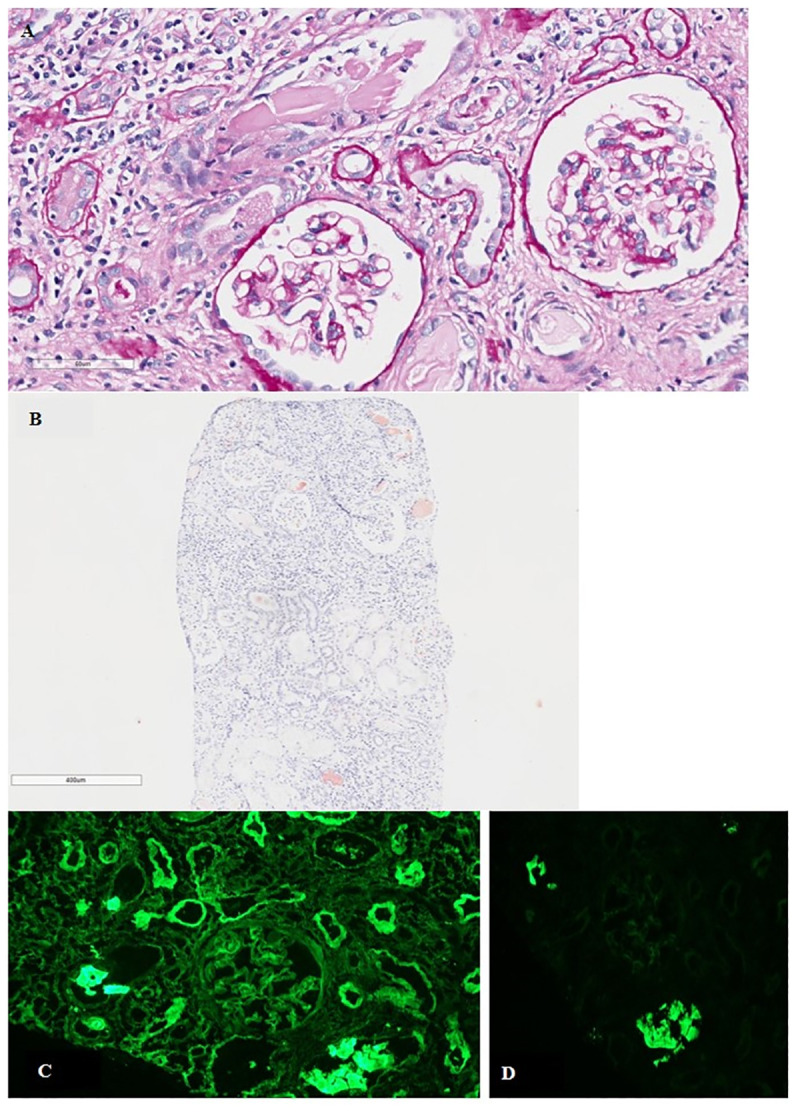
**(A)** Tubulo-interstitial nephritis with fractured intratubular cast surrounded by granulomatous inflammatory reaction. Mild mesangial matrix expansion in the glomeruli (PAS; original magnification, ×400). **(B)** Tubular casts positive for Congo red staining (original magnification, ×400). **(C)** Direct immunofluorescence for κ- and λ-LC showing κ-LC restriction along the glomerular basement membranes and tubular basement membranes (original magnification, ×200). **(D)** Polyclonal reaction in tubular casts (original magnification, ×200). *PAS*, periodic acid–Schiff stain; *LC*, light chain.

In August 2023, a 41-year-old man without relevant medical history had asymptomatic documentation (during routine laboratory exams) of severe anemia (hemoglobin = 6.8 g/dl) and AKI [creatinine = 7 mg/dl, estimated glomerular filtration rate (eGFR) = 9 ml/min]. Further laboratory tests (**Table 1**) revealed the presence of nephrotic-range proteinuria of 3.6 g/day (2.9 g/day of monoclonal kappa light chain, κ-LC), κ-sFLC of 32,970 mg/L (κ/λ ratio = 1,490), and a positive serum immunofixation for κ-LC (not quantifiable by serum protein electrophoresis), leading to suspicion of MM, likely with LCCN. Bone marrow biopsy confirmed the diagnosis of MM with clonal PCs 35% (CD138^+^PAX5^−^) and showed the co-presence of an indolent non-Hodgkin’s B-cell lymphoma (NHL) (B-lymphoid cells 15%, CD20^+^PAX5^+^CD19^+^CD5^−^CD138^−^; PC differentiated lymphocytes 5%, CD138^+^PAX5^+^) harboring the *MYD88* L265P mutation (identified via Sanger sequencing). Fluorescence *in situ* hybridization (FISH) analysis of the PCs was negative for chromosome 14 translocations, chromosome 17 deletion, or chromosome 1 abnormalities, defining a cytogenetically standard-risk disease (9). The International Staging System (ISS) was III (β2-microglobulin = 22 mg/L), the Revised ISS (R-ISS) was II, and R2-ISS was III. Skeletal assessment through ^18^F-FDG-PET/CT documented bone marrow diffuse uptake without osteolytic or focal lesions (FLs) and did not show lymphadenopathies or organomegaly. Magnetic resonance imaging (MRI) of the axial skeleton showed diffuse hypointense signal on T1-weighted sequences without FLs. Despite the high probability of LCCN, renal biopsy was performed to confirm the etiology of AKI and to assess the entity of the kidney injury and the interstitial compartment. A histological picture of diffuse acute and chronic interstitial nephritis was found, associated with moderate fractured, metachromatic with giant cell reaction casts restricted for κ-LC on the immunofluorescence studies consistent with LCCN and Congo red positivity suggestive of amyloid. In addition, diagnostic features of LCDD were found in the glomeruli, with mild mesangial matrix expansion, Congo red negativity, and κ-LC restriction on immunofluorescence. Furthermore, a mixed interstitial infiltrate of lymphocytes and PCs, both exhibiting κ-LC restriction, was observed (**Figure 1**). A transthoracic echocardiogram described mild thickening of the ventricular walls [interventricular septum (IVS), thickness of 1.2 cm] and mild pericardial effusion, without kinetic alterations (62% ejection fraction, EF). Cardiac MRI arose suspicion of infiltrative disease; however, endomyocardial biopsy to confirm AL was not performed in order to allow rapid treatment initiation. There were neither laboratory or imaging signs of liver AL nor clinical symptoms of gastrointestinal or neurological involvement.

**Table 1:** Main laboratory findings at diagnosis, before autologous stem cell transplant (ASCT), and before maintenance.

Laboratory tests	At diagnosis	Post-induction therapy (prior to ASCT)	Prior to maintenance therapy
Hemoglobin (g/dl)	6.8	9.3	11.1
Platelets (×10^9^/L)	97	113	113
Leukocytes (×10^9^/L)	5.5	4.8	2.4
Neutrophils (×10^9^/L)	3.1	3.7	1.5
Creatinine (mg/dl)	7	4	3.3
eGFR (ml/min)	9	17	22
Calcium (mg/dl)	9.7	8.3	9.2
β2-microglobulin (mg/L)	22	11.2	8.8
Albumin (g/dl)	4.3	4	4.1
LDH (IU/L)	209	238	166
Serum immunofixation	κ-LC	κ-LC	Negative
κ-sFLC (mg/L)	32,970	1,220	130
κ/λ ratio	1,490	80	22
Urine immunofixation	κ-LC	κ-LC	κ-LC
Total urine protein (g/24 h)	3.6	0.9	0.3
Urine M-protein (g/24 h)	2.9	0.6	0.08

*eGFR*, estimated glomerular filtration rate; *LDH*, lactate dehydrogenase; *LC*, light chain; *sFLC*, serum free light chain; *M-protein*, myeloma protein

## Case description: treatment and outcome

As the severe renal damage was attributable to the PC dyscrasia, despite the concomitant presence of indolent NHL, a quadruplet anti-MM induction regimen based on daratumumab, bortezomib, thalidomide, and dexamethasone (D-VTd) was started. Concurrently, five sessions of extracorporeal removal of sFLC with high-cutoff hemodialysis were initiated in August 2023, followed by hemodialysis three times per week (10, 11).

Overall, six D-VTd induction cycles were administered between August 2023 and January 2024. Adverse events during the induction therapy included grade 1 peripheral sensory neuropathy, episodes of atrial fibrillation (likely due to the presence of a transjugular central venous catheter) requiring both pharmacological and electrical cardioversion, and deep vein thrombosis subsequent to a transfemoral catheter placed to proceed with the dialysis. Due to the thrombotic event, thalidomide was temporarily withheld, and hemodialysis was switched to peritoneal dialysis.

Disease assessment upon induction therapy showed the achievement of hematologic partial response (PR) according to the International Myeloma Working Group (IMWG) criteria (12), with urine M-protein of 0.6 g/day, positive serum immunofixation (κ-LC), and κ-sFLC of 1,220 mg/L (κ/λ ratio = 80). Furthermore, a minor renal response (10 with eGFR equal to 17 ml/min was attained (**Table 1**). Bone marrow biopsy displayed the presence of a mixed population of PCs (predominant) and lymphoid cells, representing 15% of the overall cellularity.

In the absence of cardiological contraindications, the therapeutic process continued with hematopoietic stem cell mobilization with granulocyte colony-stimulating factor and plerixafor, high-dose chemotherapy (HDT) with melphalan (100 mg/m^2^, reduced dose for renal function), and autologous stem cell transplant (ASCT) (February 2024). Adverse events of the HDT included mucositis and febrile neutropenia. During hospitalization, peritoneal dialysis was temporarily switched back to hemodialysis in order to reduce the risk of infection.

Subsequently, based on real-life/retrospective data (13), due to the clinical high-risk features, maintenance therapy with bortezomib and lenalidomide was started 3 months after ASCT (May 2024), as the presence of double hematologic disease and renal impairment prevented enrolment in clinical trials.

Pre-maintenance disease assessment documented the achievement of a hematologic very good PR (VGPR): urine M-protein of 0.08 g/day, negative serum immunofixation, and κ-sFLC of 130 mg/L (κ/λ ratio = 22) (**Table 1**). Histologic re-evaluation through bone marrow biopsy resulted negative for both MM and lymphoma. Minimal residual disease (MRD), assessed using next-generation sequencing (NGS), was positive (10^−4^ sensitivity). Sanger sequencing of MYD88 resulted wild type for L265P mutation. Cardiologic evaluation showed clinical and echocardiographic stability (IVS thickness = 1.2 cm, EF = 69%). Moreover, a minor renal response was confirmed, with further improvement of kidney function after ASCT (eGFR = 22 ml/min), leading to a progressive reduction of the dialysis weekly sessions until discontinuation 4 months after ASCT.

As of June 2025, the patient has received 12 cycles of maintenance therapy, retaining a hematologic VGPR and dialysis independence.

## Discussion and patient perspective

Notably, we herein reported on a rare case of a patient with two lymphoproliferative disorders, MM and LPL, presenting with AKI and with kidney biopsy revealing the unexpected coexistence of Congo red-positive LCCN, LCDD, and interstitial infiltrate of tumor cells.

The co-occurrence of MM and LPL has been previously described in a small number of case reports. A recent case series (14) has underlined that the two entities are not always associated with biclonal M-protein (similarly to our patient), thus emphasizing the importance of integrating clinical, morphologic, immunophenotypic, and genetic data to overcome difficult diagnostic challenges and drive therapeutic decision-making.

The uniqueness of the reported case is the concomitant presence of several different patterns of renal damage revealed by the organ biopsy. At the time of MM diagnosis, renal impairment affects up to 50% of patients, and 2%–4% of these patients require dialysis (10). Moreover, it has been reported as an independent negative prognostic factor, with a significantly higher risk of disease progression and mortality (15). Renal failure (creatinine clearance <40 ml/min or serum creatinine >2 mg/dl) in patients with MM is currently considered a MDE only when caused by LCCN (upon histological confirmation or presumptive diagnosis) as nearly all such cases are associated with a high tumor burden (1). LCCN represents the most common cause of kidney disease in MM as it is documented in 40%–60% of biopsies in MM patients with renal impairment (16). This disease is mainly attributable to the toxic effect of monoclonal sFLCs, which are overproduced and overpass the absorptive and catabolic capacity of proximal tubule cells, hence reaching the distal nephron and forming aggregates with Tamm–Horsfall protein (uromodulin). Such aggregates precipitate and result in the formation of casts determining tubular obstruction and AKI. The massive endocytosis of FLCs in the proximal tubule and the distal obstruction induced by casts also cause oxidative stress, inflammation, apoptosis, and fibrosis, which may lead to chronic damage (10, 11).

Nevertheless, even smaller PC clones and other lymphoproliferative dyscrasias may be causative of renal damage, which are mainly determined by the direct deposition within one or more renal compartments of monoclonal immunoglobulins, their parts, or heterogeneous products of aggregations or by indirect/immunological mechanisms and are currently addressed as MGRS (2). The most frequent patterns are represented by AL (primarily affecting the glomeruli, blood vessels, and, less frequently, the interstitium) and monoclonal immunoglobulin deposition disease (MIDD), particularly its subtype LCDD (involving the glomerular and tubular basement membranes) (3, 17, 18). As most MGRS affect the glomeruli, unlike LCCN, they are typically causative of high albuminuria (19). Renal biopsy represents the only tool for the diagnosis of MGRS and the identification of the specific damage pattern and is recommended in patients with monoclonal gammopathy and unexplained kidney disease, atypical clinical course in the presence of known risk factors for chronic kidney disease, monoclonal gammopathy, and kidney disease in patients <50 years (2). Conversely, renal biopsy is not considered necessary by the current IMWG recommendations in cases of MM with selective proteinuria and high sFLC (≥500 mg/L) without relevant comorbidities, as renal impairment is usually caused by LCCN in such cases (10). However, even though they are often associated with smaller clones, kidney AL and LCDD are documented in 7%–30% and 19%–26% of patients with MM and renal impairment, respectively (16). It has been hypothesized that the diverse physicochemical properties of LCs may be causative of the different renal damages, and it was shown that LCs from patients with LCCN or AL reproduce the same original pattern when injected into mice (20). The concomitant presence of heterogeneous patterns of renal injury has been described in up to 16% of patients affected by PC dyscrasias undergoing organ biopsy for renal impairment (7), with the combination of LCCN and LCDD being reported as the most common. Cases with distinct concomitant damages have been explained by several theories, including the presence of biclonal diseases, acquired mutations within the LC gene, or non-fibrillar proteins serving as precursors for fibrillar ones (6, 21).

It should be highlighted that the use of effective anti-clone treatment is essential, as the achievement of deep hematologic response is significantly associated with renal response both in MM and MGRS (3, 17, 22, 23). However, the therapies for MM (including cases presenting with LCCN) and AL are currently based on specific guidelines (10, 24–26), whereas there is no consensus regarding the management of other MGRS subtypes due to the lack of data and patients receive heterogeneous treatment options (3, 17).

According to the IMWG criteria, the renal response in MM is based on the best creatinine clearance achieved (10) and is observed in approximately 70% of patients with LCCN, with the rapid reduction of sFLCs representing the most important factor for renal recovery (10, 23). Conversely, the renal responses in AL and other MGRS are based on both the eGFR and the reduction of proteinuria (27, 28) and are achieved in 46%–59% of patients (with discordant data on the differences between AL and non-AL MGRS) (3, 17). A multicenter retrospective study on patients with LCCN demonstrated that the concomitant presence of LCDD or AL (in 6.2% and 2.2% of patients, respectively) is associated with a lower probability of renal response compared with LCCN only (23). Furthermore, another retrospective analysis comparing the clinical and renal outcomes of patients with LCCN, LCDD, and their combination showed inferior overall survival in the latter group compared with LCDD alone, and similar to that of LCCN alone, whereas the death-censored renal survival was similar among the three groups. Half of the patients with both LCNN and LCDD remained dialysis-dependent, and AKI at presentation was documented as the most important renal prognostic factor (29). In our patient, the anti-MM therapy resolved the nephrotic-range proteinuria and slowly improved and stabilized the kidney function, allowing the discontinuation of dialysis after ASCT.

Moreover, renal biopsy revealed the presence of focal intratubular amyloid without evidence of glomerular, vascular, or interstitial amyloid deposits. This scenario was observed in 28% of LCCN cases and resulted significantly associated with the occurrence of systemic AL in a retrospective study (30).

Furthermore, the presence of a mixed infiltrate of lymphocytes and PCs was documented in our patient. MM infiltration into the renal parenchyma has been mainly described in heavily pretreated patients (reported in up to 29% of autopsy series) (31), while it is considered a rare event in newly diagnosed ones, with unclear clinical and renal prognostic significance. However, potential contributes to renal damage (likely due to the compression of tubules and microvasculature, local LC, and cytokine-mediated injury) has been hypothesized (32). Similarly, kidney involvement in NHL has been reported in up to one-third of patients at autopsies, but is considered rarely associated with renal impairment. Nevertheless, a recent retrospective experience regarding patients with both aggressive and indolent NHL, including LPL, undergoing renal biopsy (for AKI, chronic kidney disease, or proteinuria) has documented the presence of lymphomatous renal involvement in 85% of patients, with interstitial infiltrate being the most common lesion and only 38% of patients having radiological anomalies (33).

Overall, our case showed the unique co-occurrence of different histological patterns of kidney damage in a patient concomitantly affected by two lymphoproliferative disorders. According to the IMWG recommendations, renal biopsy would not be necessary in the diagnostic workup of our patient as the presentation with AKI, the elevated sFLC burden, and the monoclonal proteinuria would allow a presumptive diagnosis of LCCN. However, kidney biopsy also revealed the concomitant unexpected presence of an extramedullary tumor spread, LCDD, and focal intratubular amyloid within casts. Although these findings did not lead to changes regarding the initial treatment choices, they allowed a more refined prognostic stratification due to the evidence of extramedullary disease and of the co-occurrence of other histological damages beyond LCCN, likely explaining the improvement without full recovery of the renal impairment. Furthermore, renal biopsy unveiled the presence of specific damages potentially associated with extra-renal involvement, particularly cardiac infiltrative disease, although a confirmatory biopsy was not performed. This case highlights the complex heterogeneous etiologies of renal disease in patients with hematologic malignancies and emphasizes the fundamental role of kidney biopsy in improving diagnosis, comprehension of the etiology and physiopathology of the renal impairment, and, hopefully, treatment approaches.

## Data Availability

The original contributions presented in the study are included in the article/supplementary material. Further inquiries can be directed to the corresponding author.
